# Impact of Educational Intervention on Anticoagulation Control Using SAMe-TT_2_2R_2_ Score-Guided Strategy in Atrial Fibrillation

**DOI:** 10.1016/j.jacasi.2024.08.011

**Published:** 2024-10-08

**Authors:** Arintaya Phrommintikul, Surakit Nathisuwan, Wanwarang Wongcharoen, Rungroj Krittayaphong, Siriluck Gunaparn, Antika Wongthanee, Jonathan Mathers, Sue Jowett, Kate Jolly, Deirdre A. Lane, G. Neil Thomas, Gregory Y.H. Lip, TREATS-AF Study Group, Arintaya Phrommintikul, Gregory Y.H. Lip, G Neil Thomas, Rungroj Krittiyaphong, Wanwarang Wongcharoen, Surakit Nathisuwan, Gemma Slinn, Sukhi Sehmi, Siriluck Gunaparn, Deirdre Lane, Kate Jolly, Susan Jowett, Unchalee Permsuwan, Jonathan Mathers, Wichuda Jiraporncharoen, Chaisiri Angkurawaranon, Anita Slade, Samir Mehta, Antika Wongthanee, Neil Winkle, Smonporn Boonyaratavej Songmuang, Rapeephon Kunjara Na Ayudhya, Lin Yenn-Jiang, Tomorn Thongsri, Tippawan Liabsuetrakul, Kemmawadee Preedalikit, Thoranis Chantrarat, Wattana Wongtheptien, Suparat Wattanasombat, Jutarat Kaewdam, Nongluk Mananusorn, Wuttichai Sawatna, Thanyaluck Chotayaporn, Kultida Lertthanaphol, Busarakam Pothongsunun, Jirarat Chandee, Tananya Prasit, Chotika Ruttanapornnukul, Ketsanee Sripattanatrakul, Vullida Cheingyothakul, Narawudt Prasertwitayakij, Kanokporn Niwatananun, Voratima Silavanich, Mantiwee Nimworapan, Phornwinee Somsap, Tikumporn Pornwisrtsirikul, Praphaphan Daoram, Chayanit Srisongmuang, Natrawee Bureekam, Siriporn Intharangsri, Chonlada Kawila, Arjbordin Winigkul, Yodying Kaolawanich, Ajjma Sarapakdi, Usanee Pienpattapong, Olaree Chaiphet, Bancha Sookananchai, Piti Niyomsiriwanich, Weerapan Wiwatworapan, Ubonwan Sapoo, Suttiwanik Kowitphattana, Ketmanee Sae-Ueng, Arisorn Jirapermpun, Vichai Senthong, Supaporn Onsanit, Siriporn Jantharuechai, Wachiraya Tipboonchu

**Affiliations:** a Department of Internal Medicine, Faculty of Medicine, Chiang Mai University, Chiang Mai, Thailand; b Department of Pharmacy, Faculty of Pharmacy, Mahidol University, Bangkok, Thailand; c Faculty of Medicine, Siriraj Hospital, Mahidol University, Bangkok, Thailand; d Institute of Applied Health Research, University of Birmingham, Birmingham, United Kingdom; e Department of Cardiovascular and Metabolic Medicine, Institute of Life Course and Medical Sciences, University of Liverpool, Liverpool, United Kingdom; f Liverpool Centre for Cardiovascular Science at University of Liverpool, Liverpool John Moores University and Liverpool Heart & Chest Hospital, Liverpool, United Kingdom; g Danish Center for Health Services Research, Department of Clinical Medicine, Aalborg University, Aalborg, Denmark

**Keywords:** anticoagulants, atrial fibrillation, education-behavioral, SAMe-TT_2_R_2_ score, warfarin

## Abstract

**Background:**

An educational-behavioral intervention has been shown to improve anticoagulation control with warfarin in atrial fibrillation (AF) patients, but widespread application may not be practical. The SAMe-TT_2_R_2_ score was formulated to identify the likelihood of achieving optimal time in therapeutic range (TTR).

**Objectives:**

The authors conducted a randomized controlled trial to evaluate the impact of a SAMe-TT_2_R_2_ score-guided strategy for an educational-behavioral intervention, compared with usual care on patient’s anticoagulation control.

**Methods:**

Anticoagulant-naive adult AF patients were randomized to a SAMe-TT_2_R_2_ score-guided strategy or usual care. In the SAMe-TT_2_R_2_ score-guided strategy group, scores 0 to 2 received usual care, >2 received educational-behavioral intervention plus usual care. All received warfarin targeting international normalized ratio 2.0 to 3.0. Primary outcome was TTR at 12 months. Secondary outcomes included TTR at 6 months, thromboembolic and bleeding events, major adverse cardiovascular events at 12 months, and change in AF knowledge at 6 and 12 months.

**Results:**

A total of 320 patients (mean age 69.5 years; 48.8% female) were randomized to a SAMe-TT_2_R_2_ score-guided strategy plus usual care (n = 156) or usual care alone (n = 164). Mean CHA_2_DS_2_-VASc score and SAMe-TT_2_R_2_ score were 3.1 ± 1.4 and 3.3 ± 0.9, respectively. At 12 months, mean TTR was not significantly different between groups (41.0 [95% CI: 36.7-45.2] in the SAMe-TT_2_R_2_ score-guided strategy vs 40.2 [95% CI: 35.9-44.4] with usual care, and the difference between the 2 groups was 0.7 [95% CI: −5.2 to 6.6]). There were no significant differences in secondary outcomes.

**Conclusions:**

SAMe-TT_2_R_2_ score-guided strategy for an educational-behavioral intervention, compared with usual care did not significantly improve outcomes over 12 months. (A prospective randomised trial examining the impact of an intensive educational intervention versus usual care on anticoagulation therapy control based on SAMe-TT_2_R_2_ score guided strategy in anticoagulant-naive Thai patients with atrial fibrillation; TCTR20180711003)

Atrial fibrillation (AF) prevalence is increasing globally, including in Thailand, due to the rapid change of population dynamics towards an ageing society and comorbidities,^1^^,^^2^ leading to a greater health care burden associated with increased mortality and morbidity associated with AF from stroke, heart failure, and dementia, as well as hospitalizations and increasing health care costs.^3^, ^4^, ^5^

Oral anticoagulation (OAC) either with a vitamin-K antagonist (VKA) (eg, warfarin) or direct oral anticoagulants (DOAC) in eligible patients, is one of the pillars of AF management for stroke prevention. Although the DOACs have various advantages over VKA and are the preferred OAC in eligible patients with AF, access to DOACs are limited in low-to-middle income countries due to the cost.^6^^,^^7^ In Thailand, warfarin remains the standard of care, but is often suboptimally managed with the average time in therapeutic range (TTR) about 50% to 55%.^8^, ^9^, ^10^ Those with suboptimal anticoagulation control have been associated with significantly increased risk of thromboembolism, major bleeding, and death.^10^ Therefore, strategies to improve TTR and anticoagulation care are essential to improve outcomes in patients with AF, thereby impacting health care costs.^5^

There are numerous modifiable and non-modifiable factors affecting TTR including poor medication adherence, and drug, alcohol, or diet interactions with warfarin.^11^ Also, patients’ lack of knowledge and understanding of the risks/benefits of OAC may contribute to suboptimal TTR.^12^ In the TREAT (TRial of an Educational intervention on patients’ knowledge of Atrial fibrillation and anticoagulant therapy, INR control, and outcome of Treatment with warfarin) study, a one-off educational-behavioral intervention for warfarin in patients with AF (TREAT intervention) significantly improved TTR at 6 months after warfarin initiation compared with usual care alone.^12^, ^13^, ^14^ However, widespread application of an educational-behavioral intervention in every AF patient receiving warfarin may not be practical. Appropriately identifying patients who are less likely to achieve good international normalized ratio (INR) control and provide a 1-time structured educational-behavioral intervention may be a simple and cost-effective adjunct management strategy.

The SAMe-TT_2_R_2_ score, which includes demographic and clinical variables, sex (female), age (<60 years), medical history (at least 2 of the following: hypertension, diabetes mellitus, coronary artery disease/myocardial infarction, peripheral arterial disease, congestive heart failure, previous stroke, pulmonary disease, hepatic or renal disease), treatment with interacting drug, tobacco used (within 2 years), and race (non-Caucasian), was formulated to identify likelihood of achieving optimal TTR.^15^ Patients with SAMe-TT_2_R_2_ scores 0 to 2 are more likely to achieve and maintain optimal TTR, whereas those with a SAMe-TT_2_R_2_ score >2 are likely to be poorer responders, as shown in recent observational cohorts.^11^^,^^16^^,^^17^

We, therefore, conducted a randomized controlled trial in anticoagulant-naive Thai patients with AF to evaluate the impact of a SAMe-TT_2_R_2_ score-guided strategy for an educational-behavioral intervention (plus usual care), compared with usual care alone on the patient’s anticoagulation control with warfarin, as measured by the TTR at 12 months. The impact of this SAMe-TT_2_R_2_ score-guided intervention on TTR at 6 months, as well as patient knowledge, thromboembolic and bleeding events, and the composite of major adverse cardiovascular events (MACE) were evaluated as secondary objectives.

## Methods

### Study design and patient population

This was a multicenter, open-label, parallel-group, randomized controlled trial conducted among adult Thai patients (age ≥18 years) with AF who were eligible for anticoagulation (CHA_2_DS_2_-VASc ≥1 for men and ≥2 for women), but were anticoagulant-naive at baseline (no treatment with anticoagulation within the past 12 months, although treatment may have started within the 28 days before randomization), and able to comply with scheduled visits, treatment plan, and study protocol (with support of a carer). The study design, full inclusion, and exclusion criteria of the TREATS-AF have been described elsewhere.^18^

Patients with the following conditions were excluded from the study: 1) any contraindication to OAC; 2) prosthetic cardiac valve or significant valvular heart disease with an indication for heart surgery; 3) likelihood of intermittent or permanent discontinuation of warfarin during follow-up; 4) known active malignancy with a life expectancy <5 years; 5) diagnosed significant cognitive impairment preventing provision of informed consent and/or able to comply with the study protocol; and 6) any disease likely to cause death within 12 months.

All patients received warfarin, dose-adjusted to achieve a target INR of 2.0 to 3.0. INR monitoring was undertaken at routine intervals by anticoagulation services and who were blinded to treatment allocation.

The study has been approved by the Ethical Review Committee, Ministry of Public Health of Thailand (Approval Number COA-CREC 007/2020), Thai Clinical Trials Registry (TCTR) under the identification number TCTR20180711003.

### Randomization

Patients were randomized to 1 of 2 groups, an educational-behavioral intervention plus usual care, or to usual care alone, in a 1:1 ratio using a web-based platform. Group 1 was the usual care only group. For group 2, the patients were divided based on baseline SAMe-TT_2_R_2_ score into 2 groups, that is, group 2a patients with a SAMe-TT_2_R_2_ score 0 to 2 who received usual care only; and group 2b patients with SAMe-TT_2_R_2_ score >2 group, who received the TREAT education-behavioral intervention as an adjunct to their regular INR monitoring, to improve their TTR. Randomization were stratified based on center, sex, and baseline SAMe-TT_2_R_2_ score (0-2, 3-5, and 6-8).

### Trial intervention

The TREAT intervention is a patient-centered intervention for patients with AF codeveloped through the integration of theoretical and clinical frameworks, and patient feedback.^12^^,^^13^ This behavior-change intervention package consists of an educational booklet, diary, worksheet, and a DVD for reinforcement. The educational content covers AF causes and consequences, warfarin and INR monitoring, and lifestyle changes, as well as common barriers to optimal anticoagulation. In addition, a self-monitoring diary including diet, alcohol intake, warfarin regimen, and INR results was also provided. The TREAT intervention was translated and culturally adapted, tailored to the Thai setting. Translation involved both forward and backward translation by 2 bilingual experts. A preliminary evaluation of the Thai-adapted TREAT intervention was carried out among patients diagnosed with AF, encompassing various educational backgrounds and age groups. Patient input was integrated to enhance the usability of the Thai version of the TREAT intervention.^18^ A training session was systematically performed to instruct all study site coordinators (pharmacists and nurses) how to effectively implement the intervention. The TREAT intervention was delivered within 4 weeks of initiating warfarin. The homogeneity of education was ensured by training and video monitoring during education delivery to the participants. The patient booklets were checked during visits. The intervention fidelity was maintained through direct observation of randomly selected sessions at each center by the trainer. Patient food and dietary behaviors were assessed using their diaries during the study visit. The patients assigned to the usual care group received standard education from health care professionals (primary physicians and pharmacists) regarding AF, the necessity of warfarin treatment, and warfarin management utilizing the standard warfarin education booklet. This standardized warfarin management program has been implemented as routine care in Thailand over an extended period.

### Outcomes

The primary outcome was TTR at 12 months, determined by the Rosendaal method. Secondary outcomes included: 1) TTR at 6 months; 2) thromboembolic and bleeding events at 12 months; 3) composite MACE including nonfatal myocardial infarction, nonfatal stroke and cardiovascular death, and all-cause mortality, in an exploratory analysis (combined and individually) at 12 months; and 4) change in patients’ knowledge of AF from baseline to 6 months and 12 months, assessed by the AF knowledge questionnaire.^18^ The AF knowledge questionnaire was translated from the English version to Thai before conducting a test run among various levels of educational background. The questionnaires were completed by the patient. For those with literacy problems, the questionnaires were read out to the participant, and their responses were recorded by the staff member administering the questionnaires. Thromboembolic event was defined as new-onset neurological symptoms with diagnosis of stroke or transient neurological deficit or systemic embolism. Major bleeding was defined as bleeding type 3 to 5 according to Bleeding Academic Research Consortium (BARC).

### Sample size and power calculations

The sample size was calculated based on the mean TTR in Thai patients, which was in the range of 50% to 55% ± 26%^9^^,^^10^ and assumed a 10% difference in 12-month TTR between groups, with 90% power and a 5% significance level. The estimated sample size was 144 per arm, and after adjusting for a 10% attrition/loss to follow-up rate, a total sample size of 320 patients or 160 patients per arm was required.

### Statistical analysis

The statistical software package STATA version 16.2 (StataCorp) was used for statistical analysis. Descriptive statistics were presented for baseline demographic and clinical information. For all major outcomes, summary statistics and differences between groups were presented with CIs. Categorical variables were reported as counts with percentages. Continuous variables were summarized by the number of participants, mean ± SD, or median and IQR as appropriated. All data were analyzed by intention-to-treat principle. The primary and secondary outcomes between 2 groups were compared. Intervention effects between groups for all outcomes were adjusted for the stratification variables (center, sex, and baseline SAMe-TT_2_R_2_ score). The analysis was conducted as adjusted for the stratification variables by using a mixed effects linear regression model for continuous outcome and a mixed effects binomial regression model for binary outcome. Two-tailed *P* values <0.05 were considered statistically significant.

## Results

**Figure 1:**
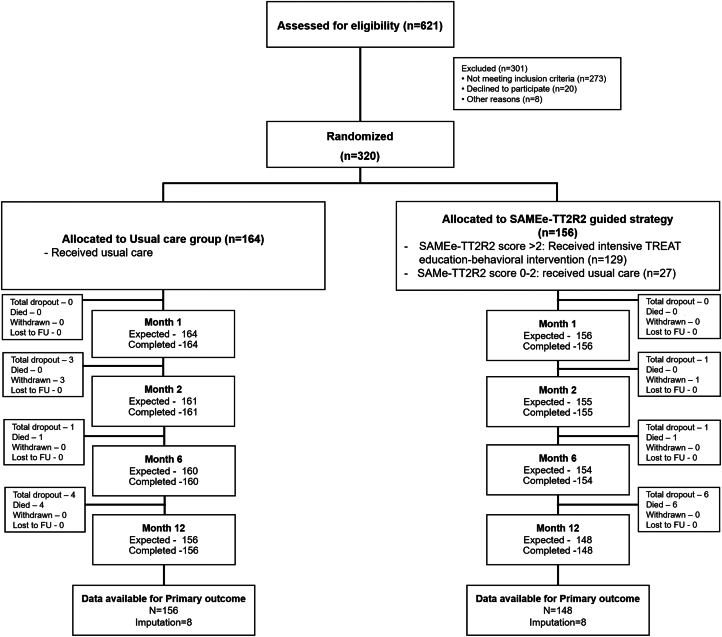
Consort Flow Diagram Atrial fibrillation patients were randomized to SAMe-TT_2_ R_2_ score-guided strategy plus usual care vs usual care alone. Patients assigned to the SAMe-TT_2_ R_2_ group who had a SAMe-TT_2_ R_2_ score 0 to 2 received usual care, whereas those who had SAMe-TT_2_ R_2_ >2 received TREAT intervention plus usual care. FU = follow-up.

From 621 patients with AF who were screened and assessed for eligibility, 273 patients (43.9%) did not meet inclusion criteria, 20 patients (0.3%) declined participation, and 8 patients (1.3%) were excluded for other reasons (Figure 1). A total of 320 patients (mean age 69.5 ± 10.2 years; 156 [48.8%] female) were enrolled and randomized to either usual care alone (n = 164) or a SAMe-TT_2_R_2_ score-guided strategy plus usual care (n = 156). The median (Q1-Q3) duration of AF before enrolment was 7.5 (Q1-Q3: 1-30) days, and paroxysmal AF was present in 41 (12.8%). Mean CHA_2_DS_2_-VASc score was 3.1 ± 1.4, and mean SAMe-TT_2_R_2_ score was 3.3 ± 0.9. The proportions of patients with a SAMe-TT_2_R_2_ score of 0 to 2 or 3 to 8 were 54 (16.9%) and 266 (83.1%), respectively. The clinical characteristics were comparable between groups at baseline (Table 1). Following randomization, all of the patients assigned to the SAMe-TT_2_R_2_ score-guided strategy with SAMe-TT_2_R_2_ score >2 received the TREAT intervention within 4 weeks.

**Table 1:** Patients Demographic and Clinical Characteristics at Baseline, Overall and by Treatment Group

	Total (N = 320)	SAMe-TT_2_R_2_ Score-Guided Strategy Plus Usual Care (n = 156)	Usual Care Only (n = 164)	*P* Value
Age, y	69.5 ± 10.2	70.1 ± 10.11	69.0 ± 10.3	0.361
Female	156 (48.8)	77 (49.4)	79 (48.2)	0.832
Education				
None	65 (20.3)	36 (23.1)	29 (17.7)	0.303
Elementary/high school	192 (60.0)	87 (55.8)	105 (64.0)	
College/university	63 (19.7)	33 (21.2)	30 (18.3)	
Body mass index, kg/m^2^	24.3 ± 5.3	24.1 ± 5.6	24.5 ± 4.9	0.466
Systolic blood pressure, mm Hg	129.6 ± 19.6	130.0 ± 19.6	129.1 ± 19.6	0.683
Diastolic blood pressure, mm Hg	74.5 ± 13.6	73.9 ± 13.2	75.1 ± 14.0	0.434
Heart rate, beats/min	93.8 ± 29.1	94.9 ± 30.3	92.7 ± 28.0	0.490
Renal function: eGFR, mL/min/m^2^	65.6 ± 25.3	64.0 ± 26.7	67.0 ± 24.0	0.442^a^
Duration of known AF, mo	4.1 ± 14.7	4.1 ± 15.1	4.1 ± 14.3	0.654^a^
Congestive heart failure	72 (22.5)	31 (19.9)	41 (25.0)	0.272
Hypertension	235 (73.4)	116 (74.4)	119 (72.6)	0.716
Age 65-74 y	89 (27.8)	49 (31.4)	40 (24.4)	0.515
Age ≥75 y	75 (23.4)	39 (25.0)	36 (21.9)	0.607
Diabetes mellitus	80 (25.0)	42 (26.9)	38 (23.2)	0.438
Stroke/TIA	34 (10.6)	18 (11.5)	16 (9.8)	0.605
CHA_2_DS_2_-VASc score	3.1 ± 1.4	3.2 ± 1.4	3.0 ± 1.4	0.262
SAMe-TT_2_R_2_ score	3.33 ± 0.9	3.34 ± 0.9	3.31 ± 1.0	0.555^a^
SAMe-TT_2_R_2_ score group				
0-2	54 (16.9)	27 (17.3)	27 (16.5)	0.840
>2	266 (83.1)	129 (82.7)	137 (83.5)	
Smoking status				
Nonsmoker	230 (71.9)	114 (73.1)	116 (70.7)	0.483
Ex-smoker	68 (21.3)	34 (21.8)	34 (20.7)	
Current smoker	22 (6.9)	8 (5.1)	14 (8.5)	
Alcohol	16 (5.0)	8 (5.1)	8 (4.9)	0.918
Chronic liver disease	9 (2.8)	3 (1.9)	6 (3.7)	0.348
Chronic kidney disease	83 (25.9)	42 (26.9)	41 (25.0)	0.695
Lung disease	15 (4.7)	8 (5.1)	7 (4.3)	0.716
Amiodarone	7 (2.2)	5 (3.2)	2 (1.2)	0.225
Antiplatelets	69 (21.6)	34 (21.8)	35 (21.3)	0.921
Beta-blocker	189 (59.1)	93 (59.6)	96 (58.5)	0.844
Calcium-channel blocker	20 (6.3)	11 (7.1)	9 (5.5)	0.564
Digoxin	15 (4.7)	6 (3.9)	9 (5.5)	0.487
ACEI/ARB	131 (40.9)	57 (36.5)	74 (45.1)	0.119
Statin	190 (59.4)	96 (61.5)	94 (57.3)	0.442
Diuretic agents	67 (20.9)	30 (19.2)	37 (22.6)	0.464

Values are mean ± SD or n (%).

ACEI = angiotensin-converting enzyme inhibitor; AF = atrial fibrillation; ARB = angiotensin receptor blocker; BMI = body mass index; DBP = diastolic blood pressure; SBP = systolic blood pressure; TIA = transient ischemic attack.

a 2-sample Wilcoxon rank sum (Mann-Whitney) *U* test.

Due to the number of participants between the 2 groups not being equal, a mixed effects linear regression model was employed to adjust for differences in group sizes. Stratification variables, sex, and baseline SAMe-TT2R2 score were treated as fixed effects with the exception of randomizing center, which was treated as a random effect.

During the 12-month follow-up period, 21 patients (6.5%) died, and 4 (1.2%) withdrew from the study because they were not able to complete follow-up as per protocol. There were 156 (100%) and 148 (90.2%) patients in the SAMe-TT_2_R_2_ score-guided strategy (plus usual care) and the usual care only group, respectively, who reached the primary outcome time point (Table 2). At 12 months, the adjusted mean TTR was not significantly different between the groups (41.0% [95% CI: 36.7%-45.2%] in the SAMe-TT_2_R_2_ score-guided strategy plus usual care vs 40.2% [95% CI: 35.9%-44.4%] with usual care alone). The difference in means between the 2 groups along was 0.7 (95% CI: −5.2 to 6.6; *P* = 0.81) (Table 2). There was also no significant difference in TTR between groups at 6 months (34.1% [95% CI: 29.7%-38.5%] vs 34.8% [95% CI: 30.2%-39.3%]; *P* = 0.77 in the SAMe-TT_2_R_2_ score-guided strategy plus usual care and usual care alone groups, respectively) (Table 2). The difference in means between the 2 groups was 0.9 (95% CI: −7.1 to 5.3); *P* = 0.77. To examine the possible impact of missing data on the results, sensitivity analysis was performed on the primary outcome measure to assess the possible impact of any missing data. There was no significant difference in primary outcomes between 2 groups using intention-to-treat analysis, imputation, and complete case analysis (Supplemental Table 1).

**Table 2:** Primary and Secondary Outcomes of Participants Enrolled in the TREATS-AF Study by Study Group

	SAMe-TT2R2 Score-Guided Strategy Plus Usual Care (n = 156)	Usual Care Only (n = 164)	Adjusted Difference Between 2 Groups (95% CI)	*P* Value
Primary outcome				
TTR (%) at 12 mo^a^	41.0 (36.7-45.2)^a^	40.2 (35.9-44.4)^a^	0.7 (−5.2 to 6.6)	0.81
Secondary outcomes				
TTR at 6 mo	34.1 (29.7-38.5)^a^	34.8 (30.2-39.3)^a^	−0.9 (−7.1 to 5.3)	0.77
12-mo outcomes				
Thromboembolic events	4 (2.6)	2 (1.2)	1.5 (−1.6 to 4.6)	0.34
Major bleeding	7 (4.5)	9 (5.5)	−0.9 (−6.6 to 4.8)	0.75
Composite major adverse cardiovascular events	10 (6.4)	7 (4.3)	2.4 (−2.8 to 7.5)	0.37
Cardiovascular death	6 (3.8)	5 (3.0)	0.9 (−3.1 to 4.9)	0.66
Noncardiovascular death	7 (4.5)	3 (1.8)	2.7 (−1.2 to 6.5)	0.17
All-cause death	13 (8.3)	8 (4.9)	3.59 (−1.8 to 9.0)	0.19
Change in patients’ knowledge of AF from baseline to 6 mo	0.4 (0.1-0.7) (n = 138)	0.4 (0.1-0.7) (n = 144)	0.0 (−0.4 to 0.5)	0.88
Change in patients’ knowledge of AF from baseline to 12 mo	0.5 (0.1-0.8) (n = 126)	0.6 (0.3-0.9) (n = 134)	−0.1 (−0.6 to 0.4)	0.66

Values are mean (95% CI) or n (%).

AF = atrial fibrillation; TTR = time in therapeutic range.

a Combined results using Rubin’s rule.

The sensitivity analysis after excluding the patients with SAMe-TT2R2 score 0 to 2, there was no significant difference in TTR at 12 months between the SAMe-TT_2_R_2_ score-guided strategy plus usual care and usual care alone groups (Supplemental Table 2).

The effects of patients’ education levels on the primary outcomes were also explored. There were no significant effects of education level on the primary outcome (Supplemental Table 3).

The thromboembolic events during the 12-month period were not significantly different between groups: 4 patients (2.6%) vs 2 (1.2%); *P* = 0.34 in SAMe-TT_2_R_2_ score-guided strategy and usual care groups, respectively, and all thromboembolic events were strokes. Major bleeding events occurred in 7 patients (4.5%) in the SAMe-TT_2_R_2_ score-guided strategy group and 9 (5.5%) in the usual care group, respectively (*P* = 0.75). Among patients in the SAMe-TT_2_R_2_ score-guided strategy, the TTR in patients receiving TREAT intervention and usual care was 36.7% ± 26.1% and 38.0% ± 20.2%, respectively. The adjusted difference in means between the 2 groups was −4.4 (95% CI: −27.9 to 19.0); *P* = 0.71. The incidence of MACE was not significantly different between groups. Although all-cause death was numerically higher in the SAMe-TT_2_R_2_ score-guided strategy, this was mostly from noncardiovascular death with no statistically significant differences between groups.

The AF knowledge score was available in 126 (80.8%) and 134 (81.7%) patients in the SAMe-TT_2_R_2_ score-guided strategy plus usual care vs usual care alone groups, respectively. The mean ± SD/median (Q1-Q3) baseline AF knowledge score was 5.7 ± 1.9/6.0 (4.0-7.0) and 5.3 ± 1.7/5.0 (4.0-7.0) for the SAMe-TT_2_R_2_ score-guided strategy plus usual care vs usual care alone groups, respectively. Analogous figures at 12 months were 6.2 ± 1.5/6.0 (5.0-7.0) and 5.9 ± 1.8/6.0 (5.0-7.0), respectively. There was no significant difference between groups in the change in patients’ knowledge over the 12-month period.

## Discussion

Our study demonstrated that a SAMe-TT_2_R_2_ score-guided strategy plus usual care with usual care alone did not improve TTR at 6 and 12 months among anticoagulant-naive AF patients. In addition, there was no significant difference in thromboembolic events, major bleeding, MACE, or AF knowledge score (Central Illustration). Observational cohorts also reported that patients with a SAMe-TT_2_R_2_ score >2 were less likely to achieve a good TTR.^16^^,^^17^ For example, an analysis from a nationwide cohort of AF patients showed that the OR of having TTR <65% was 1.64 (95% CI: 1.33-1.95) in patients with SAMeTT_2_R_2_ ≥2.^16^ Similarly, patients with venous thromboembolism who had a SAMeTT_2_R_2_ score ≥2 had significant lower TTR level than patients with a score of 0 to 1.^17^ A previous study of the TREAT intervention in a UK setting showed improved TTR at 6 months compared with usual care alone, although the difference in TTR did not remain significant at 12 months.^12^

**Central illustration:**
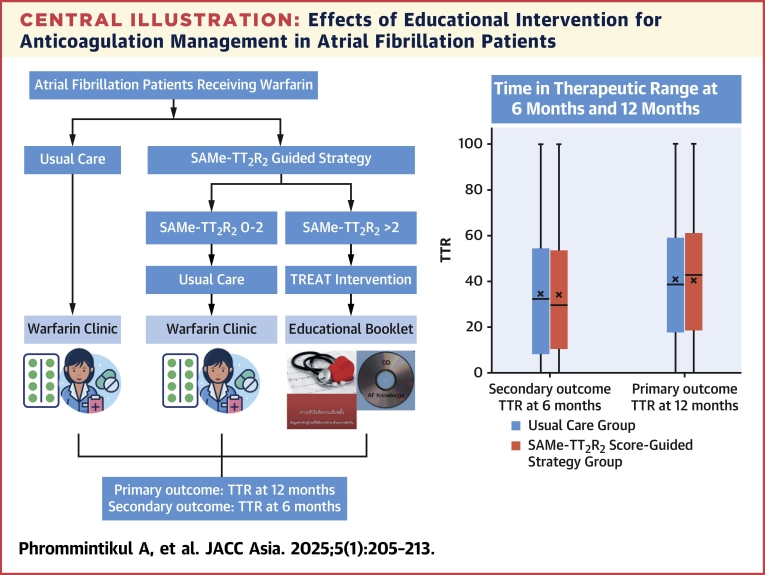
Effects of Educational Intervention for Anticoagulation Management in Atrial Fibrillation Patients Adult patients with atrial fibrillation (AF) who were eligible for warfarin were randomized to SAMe-TT_2_ R_2_ score-guided strategy vs usual care. Patients assigned to the SAMe-TT_2_ R_2_ group who had SAMe-TT_2_ R_2_ score 0 to 2 received usual care, whereas those who had SAMe-TT_2_ R_2_ >2 received TREAT intervention plus usual care. There was no significant difference in time in therapeutic range (TTR) between the 2 groups at both 6 and 12 months.

These data suggested that targeting such patients for educational intervention may improve their anticoagulation control leading us to trial the hypothesis that the TREAT intervention may improve TTR. However, in this prospective trial in OAC-eligible patients with AF, we found that the use of a SAMe-TT_2_R_2_ score-guided strategy for an educational-behavioral intervention did not lead to a significant improvement in anticoagulation control, rate of thromboembolic and bleeding events, nor was there a significant change in AF knowledge of the patients between the 2 arms.

There are some potential explanations for our findings. Although the SAMe-TT_2_R_2_ score has been tested and validated previously, these studies were mostly done in Western countries with predominantly Caucasian populations. Indeed, non-Caucasian ethnicity is identified as 1 of the factors in the SAMe-TT_2_R_2_ score predicting poor anticoagulation control.^15^ A previous observational study tested the validity of SAMe-TT_2_R_2_ score in Thai AF patients where the mean TTR at 50.%5 ± 27.5%. Results suggested that the SAMe-TT_2_R_2_ score was an independent predictor for suboptimal TTR (TTR <65%) with the C-statistic of only 0.54.^19^ Indeed, using a cutoff of the SAMe-TT_2_R_2_ score >2 to predict suboptimal TTR in Thai population may have limited sensitivity.

Although, there was no statistically significant difference in major adverse events between SAMe-TT_2_R_2_ score-guided strategy plus usual care with usual care alone. the nonsignificantly higher prevalence of history of ischemic stroke, diabetes, and hypertension may contribute to the numerically higher thromboembolic events in the SAMe-TT_2_R_2_ score-guided strategy group.

The TREAT intervention is a behavior-change intervention package consisting of an educational booklet, diary, worksheet, and a DVD for reinforcement. The format is a 1-time structured educational-behavioral intervention among those predicted to be less likely to achieve good INR control, reinforced by self-study and self-participation through documentation into the diary and worksheet. Although this intervention has been shown to be effective in Western populations, it may be less effective in a population with low health literacy though the materials had been developed with those with low health literacy. Approximately 80% of our study population has elementary school education or less, thus this could potentially be a factor for consideration. We did not observe significant improvement in the patient’s knowledge in the intervention arm, which does suggest the adaption in those with low health literacy remained suboptimal. It is also possible that the TREAT intervention was not appropriate to our patient population, and other cultural and further ethnic-specific educational interventions may need to be considered. As for the delivery of the TREAT intervention, all study site coordinators were trained on how to deliver the intervention effectively. We also prevented contamination by using separate groups of pharmacists and nurses for each arm. As a result, delivery and contamination issues should be minimized. However, due to the COVID-19 pandemic, the patient recruitment period was prolonged and may have affected the quality of intervention delivery to a certain extent. It is also important to note that the mean TTR levels in both the intervention and usual care groups (approximately 40%) were much lower than what has been reported in previous studies from Thailand (TTR approximately 50%).^10^ This can be attributed to several factors, particularly the demographic characteristics of our patient cohort, characterized by higher SAMe-TT2R2 scores and a greater prevalence of patients with SAMe-TT2R2 >2. Furthermore, the trial was conducted during the COVID-19 pandemic, leading to reduced patient visits and less frequent monitoring of INR levels. There are several factors contributing to low TTR such as genetic polymorphisms of vitamin K metabolism enzyme, and food–drug and drug–drug interactions. Alongside educational interventions, self-monitoring and self-management of warfarin represent potential strategies to enhance anticoagulation control. The integration of mobile technology may further support these approaches, providing patients with accessible and effective means of managing their warfarin therapy, particularly during periods when hospital access is restricted. Future research should focus on optimizing these interventions and evaluating their long-term impact on patient outcomes.

### Study limitations

The adaptation of the education-behavioral intervention to patients with different education levels may affect the effectiveness of the intervention. Even though the TREAT intervention was culturally adapted and test run among patients with a range of educational abilities, patients in real-world practice may be different from the enrolled patients. The generalizability to different local populations with different education levels should be considered.

One important factor that may play a role is the fact that the trial was conducted during the height of the COVID-19 pandemic. Travel restriction, lockdown, changes in lifestyle, and reassessment of financial and social priorities, may inadvertently adversely affect anticoagulation in both groups, particularly the intervals for out-patient visits, and INR tests may be suboptimal, leading to the lower TTR. Attention drawn by the pandemic fear may also adversely affect the adherence to TREAT intervention in the intervention group.

As poor TTR has been associated with more thromboembolism and bleeding, we would assume that an intervention aimed to improve TTR may impact clinical outcomes, as suggested by our prior observational cohort.^20^ Given that the TREAT intervention was insufficient to improve knowledge and anticoagulation control in our study population, this likely explains the lack of differences in the secondary outcomes, that is, thromboembolic and bleeding events, and the composite outcome between the trial arms. Additionally, the sample size was determined based on a 10% anticipated difference between the 2 groups and a desired statistical power of 90%. However, with the current sample size and no significant difference in the primary outcome being observed between the 2 groups, this resulted in very low statistical power. The post hoc power analysis for primary outcome was calculated and is presented in Supplemental Table 4.

## Conclusions

Among anticoagulant-naive Thai patients with AF, a one-off educational-behavioral intervention based on a SAMe-TT_2_R_2_ score-guided strategy plus usual care did not significantly improve anticoagulation control, patient knowledge of AF, or clinical outcomes compared with usual care alone at 12 months. Given that the study population was one with a low health literacy background, different strategies with further cultural and ethnic-specific educational interventions may be needed.

## Funding Support and Author Disclosures

This study is supported by the Newton Fund through the collaboration of the Medical Research Council (MRC; MR/R020892/1), the United Kingdom, and the Thailand Research Fund (TRF; DBG6180009), Thailand. Dr Phrommintikul has received speaker fees from Bayer, Pfizer, Boehringer Ingelheim, and Daiichi-Sankyo outside the submitted work. Dr Wongcharoen has received speaker fees from Bayer, Pfizer, Boehringer Ingelheim, and Daiichi-Sankyo outside the submitted work. Dr Lane has received investigator-initiated educational grants from Bristol Myers Squibb (BMS) and Pfizer; has received speaker fees for Boehringer Ingelheim, Bayer, and BMS/Pfizer; and has been a consultant for BMS/Pfizer and Boehringer Ingelheim, all outside of the submitted work. Prof Lip has received institutional consultancy and speaker fees for BMS/Pfizer, Boehringer Ingelheim, and Daiichi-Sankyo. All other authors have reported that they have no relationships relevant to the contents of this paper to disclose.

## References

[1] Chugh S.S., Havmoeller R., Narayanan K. (2014). Worldwide epidemiology of atrial fibrillation: a Global Burden of Disease 2010 Study. Circulation.

[2] Phrommintikul A., Detnuntarat P., Prasertwitayakij N., Wongcharoen W. (2016). Prevalence of atrial fibrillation in Thai elderly. J Geriatr Cardiol.

[3] Hindricks G., Potpara T., Dagres N. (2021). 2020 ESC guidelines for the diagnosis and management of atrial fibrillation developed in collaboration with the European Association of Cardio-Thoracic Surgery (EACTS). Eur Heart J.

[4] Ng S.S., Nathisuwan S., Phrommintikul A., Chaiyakunapruk N. (2020). Cost-effectiveness of warfarin care bundles and novel oral anticoagulants for stroke prevention in patients with atrial fibrillation in Thailand. Thromb Res.

[5] Burdett P., Lip G.Y.H. (2022). Atrial fibrillation in the UK: predicting costs of an emerging epidemic recognizing and forecasting the cost drivers of atrial fibrillation-related costs. Eur Heart J Qual Care Clin Outcomes.

[6] Krittayaphong R., Phrommintikul A., Ngamjanyaporn P. (2019). Rate of anticoagulant use, and factors associated with not prescribing anticoagulant in older Thai adults with non-valvular atrial fibrillation: a multicenter registry. J Geriatr Cardiol.

[7] Tse H.F., Wang Y.J., Ahmed Ai-Abdullah M. (2013). Stroke prevention in atrial fibrillation--an Asian stroke perspective. Heart Rhythm.

[8] Saokaew S., Sapoo U., Nathisuwan S., Chaiyakunapruk N., Permsuwan U. (2012). Anticoagulation control of pharmacist-managed collaborative care versus usual care in Thailand. Int J Clin Pharm.

[9] Priksri W., Rattanavipanon W., Saejear W. (2019). Incidence, risk factors, and outcomes of warfarin-associated major bleeding in Thai population. Pharmacoepidemiol Drug Saf.

[10] Krittayaphong R., Chantrarat T., Rojjarekampai R., Jittham P., Sairat P., Lip G.Y.H. (2020). Poor time in therapeutic range control is associated with adverse clinical outcomes in patients with non-valvular atrial fibrillation: a report from the nationwide COOL-AF registry. J Clin Med.

[11] Zulkifly H., Lip G.Y.H., Lane D.A. (2018). Use of the SAMe-TT2R2 score to predict anticoagulation control in atrial fibrillation and venous thromboembolism patients receiving vitamin K antagonists: a review. Heart Rhythm.

[12] Clarkesmith D.E., Pattison H.M., Lip G.Y., Lane D.A. (2013). Educational intervention improves anticoagulation control in atrial fibrillation patients: the TREAT randomised trial. PLoS One.

[13] Clarkesmith D.E., Pattison H.M., Borg Xuereb C., Lane D.A. (2016). Developing a complex educational-behavioural intervention: the TREAT intervention for patients with atrial fibrillation. Healthcare (Basel).

[14] Smith D.E., Xuereb C.B., Pattison H.M., Lip G.Y., Lane D.A. (2010). TRial of an Educational intervention on patients' knowledge of Atrial fibrillation and anticoagulant therapy, INR control, and outcome of Treatment with warfarin (TREAT). BMC Cardiovasc Disord.

[15] Apostolakis S., Sullivan R.M., Olshansky B., Lip G.Y.H. (2013). Factors affecting quality of anticoagulation control among patients with atrial fibrillation on warfarin: the SAMe-TT(2)R(2) score. Chest.

[16] Ruiz-Ortiz M., Bertomeu V., Cequier A., Marin F., Anguita M. (2015). Validation of the SAMe-TT2R2 score in a nationwide population of nonvalvular atrial fibrillation patients on vitamin K antagonists. Thromb Haemost.

[17] Palareti G., Antonucci E., Lip G.Y. (2016). The SAME-TT2R2 score predicts the quality of anticoagulation control in patients with acute VTE. A real-life inception cohort study. Thromb Haemost.

[18] Phrommintikul A., Nathisuwan S., Gunaparn S. (2021). Prospective randomised trial examining the impact of an educational intervention versus usual care on anticoagulation therapy control based on an SAMe-TT2R2 score-guided strategy in anticoagulant-naive Thai patients with atrial fibrillation (TREATS-AF): a study protocol. BMJ Open.

[19] Krittayaphong R., Winijkul A., Pirapatdit A. (2020). SAMe-TT2R2 score for prediction of suboptimal time in therapeutic range in a Thai population with atrial fibrillation. Singapore Med J.

[20] Lip G.Y.H., Haguenoer K., Saint-Etienne C., Fauchier L. (2014). Relationship of the SAMe-TT(2)R(2) score to poor-quality anticoagulation, stroke, clinically relevant bleeding, and mortality in patients with atrial fibrillation. Chest.

