# Supplementary material for: Impact of Educational Intervention on Anticoagulation Control Using SAMe-TT22R2 Score-Guided Strategy in Atrial Fibrillation
Source: JACC Asia. 2024 Oct 8;5(1):205–13. doi: 10.1016/j.jacasi.2024.08.011 (PMC11782003; doi:10.1016/j.jacasi.2024.08.011)
Supplement: Supplemental Material [file mmc1.docx]

**SUPPLEMENTAL APPENDIX**

**TREATS-AF Investigators**

Trial Management Group of TREATS-AF: Arintaya Phrommintikul, Gregory Y. H. Lip, G Neil Thomas, Rungroj Krittiyaphong, Wanwarang Wongcharoen, Surakit Nathisuwan, Gemma Slinn, Sukhi Sehmi, Siriluck Gunaparn, Deirdre Lane, Kate Jolly, Susan Jowett, Unchalee Permsuwan, Jonathan Mathers, Wichuda Jiraporncharoen, Chaisiri Angkurawaranon, Anita Slade, Samir Mehta, Antika Wongthanee, Neil Winkle

Trial Steering Committee – TSC: Smonporn Boonyaratavej Songmuang – Chair, Rapeephon Kunjara Na Ayudhya, Lin Yenn-Jiang, Tomorn Thongsri

Data Monitoring and Ethics Committee – DMEC: Tippawan Liabsuetrakul, Kemmawadee Preedalikit, Thoranis Chantrarat,

Participating local investigator list of TREATS-AF;

1. Chiangrai Prachanukroh Hospital: Wattana Wongtheptien, Suparat Wattanasombat, Jutarat Kaewdam, Nongluk Mananusorn, Wuttichai Sawatna

2. Nakornping Hospital: Thanyaluck Chotayaporn, Kultida Lertthanaphol, Busarakam Pothongsunun, Jirarat Chandee, Tananya Prasit, Chotika Ruttanapornnukul, Ketsanee Sripattanatrakul, Vullida Cheingyothakul

3. Maharaj Nakorn Chiang Mai Hospital: Narawudt Prasertwitayakij, Kanokporn Niwatananun, Voratima Silavanich, Mantiwee Nimworapan, Phornwinee Somsap, Tikumporn Pornwisrtsirikul, Praphaphan Daoram, Chayanit Srisongmuang

4. Lampang Hospital: Natrawee Bureekam, Siriporn Intharangsri, Chonlada Kawila

5. Siriraj Hospital: Arjbordin Winigkul, Yodying Kaolawanich, Ajjma Sarapakdi, Usanee Pienpattapong, Olaree Chaiphet

6. Maharat Nakhon Ratchasima Hospital: Bancha Sookananchai, Piti Niyomsiriwanich, Weerapan Wiwatworapan, Ubonwan Sapoo, Suttiwanik Kowitphattana, Ketmanee Sae-Ueng, Arisorn Jirapermpun

7. Srinagarind Hospital and Queen Sirikit Heart Center: Vichai Senthong, Supaporn Onsanit, Siriporn Jantharuechai, Wachiraya Tipboonchu

**Supplemental Table 1. The effects of SAMe-TT2R2 guided OAC plus usual care vs. usual care on primary outcomes (TTR at 12 months) using intention to treat analysis, imputation and complete case**

| **TTR at 12 months** | **SAMe-TT_2_R_2_ guided OAC** | **Usual care** |  |  |
| --- | --- | --- | --- | --- |
| **Intention to treat, N** |  |  |  |  |
| N | 148 | 156 | 304 |  |
| Mean (95% CI) | 41.33 (37.10 – 45.55) | 39.90 (35.63 – 44.17) | 40.59 (37.60 – 43.58) |  |
| Predictive mean (95% CI) † | 40.43 (34.22 – 46.65) | 39.36 (33.21 – 45.50) | 39.88 (34.44 – 45.32) | 1.07 (-4.80 – 6.94), p = 0.720 |
| **Imputation** |  |  |  |  |
| N | 156 | 164 | 320 |  |
| Mean (95% CI) * | 40.96 (36.71 – 45.21) | 40.16 (35.88 – 44.44) | 40.55 (37.54 – 43.56) |  |
| Predictive mean (95% CI) † | 40.16 (33.84 – 46.47) | 39.44 (33.21 – 45.67) | 39.79 (34.25 – 45.33) | 0.72 (-5.15 – 6.58), p = 0.811 |
| **Complete case** |  |  |  |  |
| N | 135 | 145 | 280 |  |
| Mean (95% CI) | 42.43 (38.06 – 46.80) | 41.62 (37.22 – 46.02) | 42.01 (38.92 – 45.09) |  |
| Predictive mean (95% CI) † | 41.51 (34.99 – 48.03) | 40.90 (34.48 – 47.32) | 41.19 (35.48 – 46.91) | 0.61 (-5.43 – 6.65), p = 0.844 |

†Adjusted by sex, baseline SAMe-TT2R2 score and center.

* Combined results using Rubin’s rule.

**Supplemental Table 2. Sensitivity analysis of primary outcome in complete case in patients with baseline** **SAMe-TT2R2 > 2**

| **TTR at 12 months** | **SAMe-TT_2_R_2_ guided OAC**  **(N=111)** | **Usual care**  **(N=121)** | **Total**  **(N=232)** | **Adj. Mean diff. (95% CI)**  **[P-value]** |
| --- | --- | --- | --- | --- |
| Mean (95% CI)  Predictive mean (95% CI) † | 42.30 (37.33 – 47.27)  41.89 (36.22 – 47.56) | 41.52 (36.80 – 46.25)  40.94 (35.44 – 46.43) | 41.89 (38.50 – 45.29)  41.39 (36.96 – 45.83) | 0.95 (-5.82 – 7.72), p = 0.783 |

†Adjusted by sex, baseline SAMe-TT2R2 score and center.

**Supplemental Table 3** Effects of education level on primary outcomes

| **Education level** | **TTR at 12 months** [Mean (95% CI), n] | | **Compare 2 treatment groups** |
| --- | --- | --- | --- |
|  | **SAMe-TT_2_R_2_ guided OAC** | **Usual care** | **Adj. Mean diff. (95% CI) †**  **[P-value]** |
| **Intention to treat** | 148 | 156 |  |
| **Total** | **41.33 (37.10 – 45.55), 148** | **39.90 (35.63 – 44.17), 156** |  |
| None | 44.94 (35.04 – 54.85), 33 | 34.58 (24.64 – 44.51), 27 | 11.41 (-1.93 – 24.74), p = 0.094 |
| Elementary/High school | 40.06 (34.05 – 46.08), 83 | 41.18 (35.71 – 46.64), 101 | -1.58 (-9.15 – 5.99), p = 0.683 |
| College/University | 40.87 (33.90 – 47.85), 32 | 40.44 (30.14 – 50.74), 28 | -0.09 (-13.47 – 13.29), p = 0.989 |
| **Test effect of education** |  |  | **Effects** |
| (Elementary/High school) & None | p = 0.859 | p = 0.046 | Treatment*Educ., p = 0.2480 |
| (College/University) & None | p = 0.987 | p = 0.129 | Treatment effect, p = 0.3477 |
| (College/University) & (Elementary/High school) | p = 0.873 | p = 0.916 | Education effect, p = 0.4159 |
| **Imputation** | 156 | 164 |  |
| **Total** | **40.96 (36.71 – 45.21), 156*** | **40.16 (35.88 – 44.44), 164*** |  |
| None | 44.25 (34.47 – 54.03), 36* | 35.63 (25.58 – 45.68), 29* | 10.02 (-3.27 – 23.31), p = 0.140 |
| Elementary/High school | 39.65 (33.63 – 45.68), 87* | 41.31 (35.85 – 46.77), 105* | -1.76 (-9.33 – 5.81), p = 0.649 |
| College/University | 40.82 (33.59 – 48.05), 33* | 40.51 (30.04 – 50.99), 30* | -0.28 (-13.79 – 13.23), p = 0.968 |
| **Test effect of education** |  |  | **Effects** |
| (Elementary/High school) & None | p = 0.867 | p = 0.071 | Treatment*Educ., p = 0.3156 |
| (College/University) & None | p = 0.996 | p = 0.170 | Treatment effect, p = 0.1396 |
| (College/University) & (Elementary/High school) | p = 0.871 | p = 0.920 | Education effect, p = 0.9786 |
| **Complete case** | 135 | 145 |  |
| **Total** | **42.43 (38.06 – 46.80), 135** | **41.62 (37.22 – 46.02), 145** |  |
| None | 43.20 (32.48 – 53.93), 29 | 38.90 (29.11 – 48.69), 24 | 5.87 (-8.21 – 19.96), p = 0.414 |
| Elementary/High school | 42.04 (35.78 – 48.30), 76 | 42.10 (36.41 – 47.80), 95 | -0.44 (-8.24 – 7.37), p = 0.913 |
| College/University | 42.67 (35.80 – 49.54), 30 | 42.35 (31.74 – 52.96), 26 | -0.14 (-13.92 – 13.63), 0.984 |
| **Test effect of education** |  |  | **Effects** |
| (Elementary/High school) & None | p = 0.741 | p = 0.192 | Treatment*Educ., p = 0.7367 |
| (College/University) & None | p = 0.716 | p = 0.273 | Treatment effect, p = 0.6242 |
| (College/University) & (Elementary/High school) | p = 0.913 | p = 0.956 | Education effect, p = 0.4916 |

†Adjusted by sex, baseline SAMe-TT2R2 score and center.

* Combined results using Rubin’s rule.

**Supplemental Table 4. Post Hoc power analysis for primary outcome**

|  | **Sensitivity method** | | | |
| --- | --- | --- | --- | --- |
|  | **Imputation** | | **Complete case** | |
|  | **SAMe-TT_2_R_2_ guided OAC** | **Usual care** | **SAMe-TT_2_R_2_ guided OAC** | **Usual care** |
| N | 156 | 164 | 135 | 145 |
| Mean | 40.96 | 40.16 | 42.43 | 41.62 |
| SEM | 2.15 | 2.17 | 2.21 | 2.23 |
| SD | 26.85 | 27.79 | 25.68 | 26.79 |
| 95% CI | 36.71 – 45.21 | 35.88 – 44.44 | 38.06 – 46.80 | 37.22 – 46.02 |
| **Adj. Mean diff. (95% CI)** | **0.72 (-5.15 to 6.58)** | | **0.61 (-5.43 to 6.65)** | |
| Alpha | 0.05 | | 0.05 | |
| **Power** | **5.78** | | **5.76** | |
